# From Individual Substances to the Broader Context of Substance Use

**DOI:** 10.1177/14550725261502844

**Published:** 2026-11-05

**Authors:** Ronja Järvelin, Aleksi Hupli

**Affiliations:** 1 Faculty of Social Sciences, 3835 University of Helsinki, Helsinki, Finland; 2 Faculty of Social Sciences, 7840 Tampere University, Tampere, Finland

In their recent article, “Might Cannabis Legalization Have Affected Public Health-Relevant Alcohol- and Tobacco-Use Outcomes in Canada?”, Fischer et al. (2026) ask how the legalization of recreational cannabis in Canada in 2018 may have affected alcohol and tobacco use. As they note, the findings are mixed and causal relationships remain unclear. Given the substantial public health harms associated with alcohol and tobacco, these potential effects warrant attention in other jurisdictions where cannabis has been legalized (Fischer et al., 2026).

Cannabis is often perceived as less harmful than alcohol (Allen et al., 2018; Unlu & Hupli, 2023). Yet there is no clear evidence that people who use cannabis generally substitute it for alcohol. Substitution has been reported in some groups, particularly among people using cannabis for medical purposes (Subbaraman, 2016). Patterns of alcohol, cannabis, and tobacco use may therefore change differently across groups (Wilkins et al., 2024). Population-level analyses may not always capture these differences. It is important to examine not only how use changes, but also among whom these changes occur and what they mean for overall substance-related harms.

The relationship between cannabis and tobacco also shows how modes of consumption can change over time. In countries where cannabis is commonly used with tobacco, such as Finland (Rönkä et al., 2023), increased cannabis use may maintain or increase tobacco smoking (Fischer et al., 2026). At the same time, the growing prevalence of cannabis vaping (Harrell et al., 2022) may alter this relationship. In Canada, for example, the proportion of people who used cannabis in the past 12 months who reported using vape pens or cartridges increased from 16% in 2018 to 37% in 2024 (Health Canada, 2025; see also Fischer et al., 2026).

Different substances are often used in combination, and people may adjust both the substances they use and their modes of consumption to achieve desired effects or reduce perceived harms (Boyle et al., 2023). Changes in substance use therefore reflect not only policy and population-level trends, but also changing cultures of use, norms, and practices of self-regulation (Sifaneck et al., 2003; Zinberg, 1984).

Fischer et al. (2026) rightly call for better population-level data and rigorous longitudinal analyses. These are needed to assess whether changes in alcohol and tobacco use are causally related to cannabis legalization. We suggest complementing this research with a stronger focus on the mechanisms behind these changes. Prevalence estimates can show what is changing and among whom. Qualitative research can help explain why these changes occur, how people understand the relationships between different substances, and how harms and benefits are experienced in everyday life. Understanding the broader effects of cannabis policy therefore requires more than identifying substitution or complementarity in substance use at the population level. It also requires examining how these relationships emerge and are experienced within different groups and cultures of use.
